# A national survey of inpatient medication systems in English NHS hospitals

**DOI:** 10.1186/1472-6963-14-93

**Published:** 2014-02-27

**Authors:** Monsey McLeod, Zamzam Ahmed, Nick Barber, Bryony Dean Franklin

**Affiliations:** 1 The Centre for Medication Safety and Service Quality, Pharmacy Department, Imperial College Healthcare NHS Trust and Department of Practice and Policy, UCL School of Pharmacy, London, UK; 2 The Health Foundation, 90 Longacre, London WC2E 9RA, UK

**Keywords:** Health care surveys, Medication systems, Pharmacy service hospital, Medication storage

## Abstract

**Background:**

Systems and processes for prescribing, supplying and administering inpatient medications can have substantial impact on medication administration errors (MAEs). However, little is known about the medication systems and processes currently used within the English National Health Service (NHS). This presents a challenge for developing NHS-wide interventions to increase medication safety. We therefore conducted a cross-sectional postal census of medication systems and processes in English NHS hospitals to address this knowledge gap.

**Methods:**

The chief pharmacist at each of all 165 acute NHS trusts was invited to complete a questionnaire for medical and surgical wards in their main hospital (July 2011). We report here the findings relating to medication systems and processes, based on 18 closed questions plus one open question about local medication safety initiatives. Non-respondents were posted another questionnaire (August 2011), and then emailed (October 2011).

**Results:**

One hundred (61% of NHS trusts) questionnaires were returned. Most hospitals used paper-based prescribing on the majority of medical and surgical inpatient wards (87% of hospitals), patient bedside medication lockers (92%), patients’ own drugs (89%) and ‘one-stop dispensing’ medication labelled with administration instructions for use at discharge as well as during the inpatient stay (85%). Less prevalent were the use of ward pharmacy technicians (62% of hospitals) or pharmacists (58%) to order medications on the majority of wards. Only 65% of hospitals used drug trolleys; 50% used patient-specific inpatient supplies on the majority of wards. Only one hospital had a pharmacy open 24 hours, but all had access to an on-call pharmacist. None reported use of unit-dose dispensing; 7% used an electronic drug cabinet in some ward areas. Overall, 85% of hospitals had a double-checking policy for intravenous medication and 58% for other specified drugs. “Do not disturb” tabards/overalls were routinely used during nurses’ drug rounds on at least one ward in 59% of hospitals.

**Conclusions:**

Inter- and intra-hospital variations in medication systems and processes exist, even within the English NHS; future research should focus on investigating their potential effects on nurses’ workflow and MAEs, and developing NHS-wide interventions to reduce MAEs.

## Background

Systems and processes used to prescribe, supply, store, and administer medicines can have a substantial impact on medication administration errors (MAEs) in hospital inpatient settings [1-8]. Nursing staff potentially play a key role in intercepting all types of medication errors and thus preventing patient harm; however nurses are also themselves susceptible to making errors [9,10]. Inadequate nurse staffing (numbers, skill mix, knowledge and experience) is a major potential patient safety concern [11-15]. Furthermore, increasing cognitive workload during medication administration as a result of system-related factors such as interruptions has been associated with both actual and perceived risk of MAEs [16,17]. Coupled with organisational pressures to reduce costs and increasing demands on the health service, there is an urgent need to develop systems and processes to increase efficiency and support the delivery of high quality safe care. In England, health care is primarily delivered by one publicly funded organisation, National Health Service (NHS) England. However, little is known about medication systems and processes currently used within the English NHS; this presents a challenge for developing NHS-wide interventions to increase medication safety.

Unlike in the United States (US) [18,19], there has been no comprehensive national survey of hospital medication systems in English hospitals. A 1992 survey of clinical pharmacy services in UK NHS hospitals [20] reported the extent to which various activities were carried out. This survey reported that 9% of hospitals had a resident on-call pharmacist and 88% had a non-resident on-call service to provide advice and support medication supply outside of pharmacy opening hours. However, these data are now over 20 years old and various national medication safety and quality initiatives have since been introduced in NHS hospitals. These include the use of electronic prescribing and medication administration (EPMA) systems, the use of patients’ own drugs (PODs) during hospital inpatient stays, one-stop dispensing (OSD) supplies which are hospital inpatient medications labelled with administration instructions for use at discharge as well as during the inpatient stay, and patient bedside medication lockers [21-23]. These interventions evolved from the recognition of common problems across the NHS. However, the extent to which these initiatives have been implemented across English hospitals is unclear. This gap in knowledge presents a potential barrier to developing systems-based interventions to support the safety and quality of medication administration. We therefore conducted a national survey of hospital medication systems in English NHS hospitals with the aim of describing the medication administration related systems and processes used. Specifically, we wanted to summarise (1) the systems and processes used for prescribing, supply, storage, transport, and administration of medications on general medical and surgical inpatient wards; and (2) local strategies introduced with the aim of reducing MAEs.

## Methods

We conducted a national cross-sectional postal census of English NHS hospital inpatient medication systems in July 2011. At the time, the English NHS was geographically divided into 10 strategic health authorities (SHAs) [24], each responsible for overseeing local health services including one or more acute and/or foundation NHS hospital trusts, where foundation trusts have more financial and operational freedom than non-foundation trusts. There were a total of 165 acute and foundation NHS trusts; each had one or more acute or specialist hospitals.

A pre-notification letter was sent to the chief pharmacist at each trust in June 2011, followed by an invitation to complete a questionnaire relating to inpatient medical and surgical wards in their main acute hospital in July 2011. To potentially increase the response rate [25], a business-franked return envelope was provided, the chief pharmacist was encouraged to delegate questionnaire completion by a deputy if appropriate, and our contact details were included. In addition, non-responders were sent another invitation, questionnaire and business-franked return envelope in August 2011, and then an electronic reminder sent to non-responders for whom we had email addresses in October 2011.

The questionnaire (see Additional file 1) comprised two parts with questions relating to medication systems and processes in part 1, and more detailed questions relating to any electronic prescribing systems used in part 2 (data presented elsewhere) [26]. All questions (both parts) were initially piloted with a range of health care professionals, followed by 15 hospital pharmacists of varying experience across four trusts to test face validity and internal consistency. One of two researchers (MM and ZA), who were familiar with hospital medication systems, also observed each respondent as they completed the questionnaire; in order to assess content validity, respondents were asked to report any problems during completion in addition to general feedback. Part 1 of the final questionnaire incorporated a number of findings from pilot work, mainly: (1) a brief explanation to emphasise the importance of the survey was included, (2) an option for respondents to select ‘one ward’ was included in questions where ‘all wards’, ‘most wards’, ‘some wards’, ‘no ward’ and ‘not sure’ were standard options, and (3) two open questions were combined. This paper presents the data from part 1 which comprised 18 closed and one open question. The 19 questions covered five areas which reflected the objectives of this study: (A) hospital demographics, (B) prescribing and medication administration records, (C) medication ordering and supply (including pharmacy services), (D) ward-based medication storage and transport during nurses’ drug rounds, and (E) medication administration processes, policies, and guidance. Eight questions had multiple parts that involved selecting one option from six: “all wards”, “most wards”, “some wards”, “one ward”, “no ward”, or “not sure”. Where relevant, respondents were also asked to identify and rank the three most common systems or practices used in their hospital. Three other closed questions had multiple parts; these involved the respondent selecting one option from three (“yes”, “no”, “not sure”) in response to whether a specific administration-related policy or guidance was available. The open question asked about local medication safety initiatives.

All completed questionnaires returned by 1 November 2011 were included in the data analysis. Questionnaire responses were transcribed and analysed using descriptive statistics within Microsoft Excel (MM). Characteristics of respondents and non-respondents were compared using parametric and non-parametric statistical tests as appropriate for the distribution of the data concerned. Transcription of a random 20% of questionnaires was verified by ZA and discrepancies reviewed jointly and resolved; a third reviewer was available to resolve any remaining disagreements but was not required. One additional completed questionnaire received in December 2011 was excluded, as were specific questions omitted by individual respondents. For questions relating to the proportion of wards that used a specific system or process, responses for ‘all wards’ and ‘most wards’ were combined into ‘majority of wards’ in our analysis of inter-hospital variation. Intra-hospital variations were identified by reviewing responses for questions for which ‘some wards’ and ‘one ward’ was selected; responses for ‘no ward’ was described separately. We conducted additional analysis by SHA of hospitals in which EPMA systems were used in the majority of wards.

The study was approved by the relevant School of Pharmacy Research Ethics Committee in June 2011. The Joint Research Office at Imperial College London and Imperial College Healthcare NHS Trust confirmed that NHS research ethics approval was not required as the study was considered to be service evaluation.

## Results

### Overview

Overall, 100 (61%) questionnaires were returned: 57 (35%) initially and 43 (26%) after follow-up. Respondents were from 59 of 93 (63%) foundation NHS trusts and 41 of 72 (57%) acute NHS trusts. Median response rate per question was 97% (range 64-100%). Characteristics of respondent and non-respondent trusts are summarised in Table 1; no statistically significant differences were identified.

**Table 1:** Comparison of respondent and non-respondent trusts

**Trust characteristic**	**Respondents (n = 100 trusts)**	Non-respondents* (n = 65 trusts)	**Statistical analysis**
Median number of acute hospitals in trust (range)	1 (1–5)	1 (1–5)	p = 0.08; Mann–Whitney test
Median number of wards at main acute hospital (range)	25 (3 – 60)	23 (1–44)	p = 0.12; Mann–Whitney test
Services provided by main acute hospital	Adults (13) or paediatrics (1) only: 14 (14%)	Adults (2) or paediatrics (3) only: 5 (8%)	p = 0.21; Chi-square test
	Mixed: 86 (86%)	Mixed: 60 (92%)	

*Data obtained from the trust websites.

An overview of specific inpatient medication systems used on the majority of medical and surgical wards is presented in Table 2. Overall, the majority of hospitals used paper-based inpatient prescribing for the majority of medical and surgical inpatients (87% of usable responses), patient bedside medication lockers (92%), ward stock (94%), PODs (89%), and OSD (85%). Ordering medications from pharmacy staff during their ward visit was more prevalent than other methods of ordering medications; the prevalence of different ordering methods varied between 13% and 62% of usable responses. Hospitals also varied in their use of drug trolleys to store medicines (59% of usable responses) on the majority of medical and surgical wards, methods used to transport medicines during drug rounds (between 8% and 65% among four different methods), and the use of non-OSD supplies which are medicines intended for use during the inpatient stay that are labelled with the patient’s name but not with instructions for administration (50%).

**Table 2:** Key features of inpatient medication systems used on the majority of medical and surgical wards

**Systems and processes**	**Number of respondent hospitals (% of usable responses)**
Prescribing and administration record	**■ Paper versus electronic prescribing system**
	87 (87%) used paper drug charts
	13 (13%) used an EPMA system
Medication ordering and supply	**■ Methods used to order medications during pharmacy opening hours†:**
	59 (62%) via the ward pharmacy technician (during their ward visit)
	55 (58%) via the ward pharmacist (during their ward visit)
	26 (29%) via the ward pharmacist (outside of their ward visit)
	24 (26%) by taking drug charts to the pharmacy
	12 (13%) by computer/electronically
	5 (5%) selected ‘other’: ‘pneumatic tubes’ (n = 2), “pharmacy teams are ward based” (1), “bleeping [paging] the sweep pharmacist [designated to order medication across a range of wards] in the afternoon” (1), “nurse ordering” (1).
	**■ Methods used to obtain medications outside pharmacy opening hours†:**
	97 (97%) borrowed medicines from another ward
	96 (96%) contacted the on-call pharmacist
	89 (89%) used a non-electronic reserve drug cupboard
	39 (39%) borrowed from another patient’s hospital supply (on the same ward)
	11 (11%) used an electronic reserve drug cupboard
	9 (9%) selected ‘other’: asked the family to bring in PODs (n = 5), accessed a dispensing robot via the on-call pharmacist (2), medicines were not generally ordered outside of hours (1), 24-hour pharmacy (1).
	**■ Types of medication supply for inpatient administration†:**
	89 (94%) used ward stock
	85 (89%) used PODs
	82 (85%) used OSD supplies from the hospital pharmacy
	46 (50%) used non-OSD supplies from the hospital pharmacy
	3 (3%) selected ‘other’: all referred to the use of pre-labelled packs
Ward-based medication storage and transport during nurses’ drug rounds	■** Ward-based medication storage† (see also Figure **5**):**
	91 (92%) used patient bedside medication lockers
	55 (59%) used drug trolleys
	**■ Medication transport during drug rounds†:**
	64 (65%) used drug trolleys
	31 (43%) used medicines cup/oral syringe
	10 (14%) used a tray/basket
	6 (8%) used a temporary trolley (for example, dressing trolley)
	2 (2%) selected ‘other’: 1 used “PRN lockers per bay”, 1 “drugs cupboard in [each] 6-bedded bay”
Medication administration processes, policies and guidance	**■ Regularly scheduled drug rounds (99; 100%)**
	**■ Availability of policies and guidance:**
	97 (98%) had an ‘out of hours access to medications’ guidance document
	95 (97%) had guidance document on what to do if a drug was not available
	90 (93%) had a ‘patient self-administration’ policy
	80 (92%) had a ‘nil-by-mouth’ policy
	98 (99%) had an IV guide: 71 (73%) paper-based version, 81 (82%) electronic

†Percentage total was over 100 as more than one option could be selected by the respondent.

EPMA, electronic prescribing and medication administration; IV, intravenous; NHS, National Health Service; OSD, one-stop dispensing; PODs, patients’ own drugs; PRN, pro re nata or ‘when required’.

### Prescribing and medication administration records

**Figure 1:**
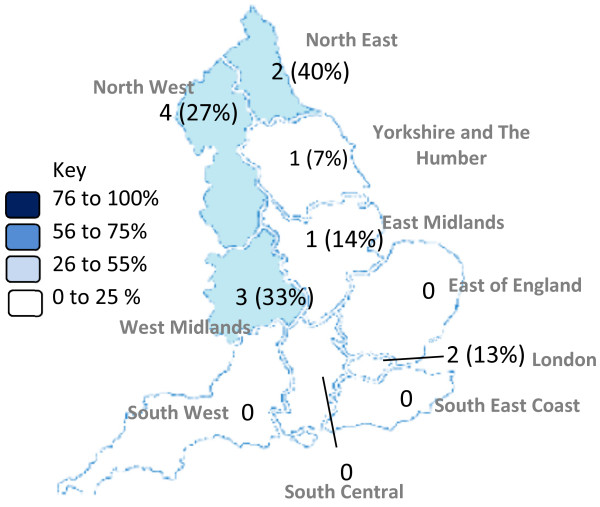
**Prevalence of inpatient electronic prescribing and medication administration (EPMA) systems in English NHS trusts, presented by strategic health authority (SHA).** Figures refer to number of trusts (percentage within each SHA) that had an EPMA system on the majority of inpatient medical and surgical wards in their main acute hospital.

The 13 hospitals in which an EPMA system was used on the majority of inpatient medical and surgical wards were mainly located in the northern SHAs (Figure 1). Exploratory analysis suggested that EPMA systems were more likely in foundation than acute trusts (13 foundation trusts versus 0 acute trusts, p = 0.001, chi-square test). Of all 100 respondent hospitals, an additional 6 (6%) also used an EPMA system on ‘one’ or ‘some’ medical and/or surgical wards; other wards in the same hospital used a paper-based inpatient prescribing system. More details of the EPMA systems used are presented elsewhere [26].

### Medication ordering and supply (including pharmacy services)

One respondent hospital had a pharmacy open 24 hours a day. Of the remaining 99 hospitals, the pharmacy was open for a median of 9 hours on weekdays (95% confidence interval 9–10 hours), 5 hours on Saturdays (95% CI 4–5 hours), and 3 hours on Sundays (95% CI 2–4 hours). The pharmacy in 3 (3%) hospitals was not open on Saturdays or Sundays, and the pharmacy in a further 26 (26%) hospitals was open on Saturdays but not Sundays. To support medication supply outside pharmacy opening hours, 90 (90%) respondent hospitals had a non-resident on-call pharmacist and 9 (9%) had a resident [on-site] on-call pharmacist. There was a non-significant trend towards hospitals with a non-resident on-call pharmacist being smaller (mean 25 wards per hospital, 95% CI 23–28) than those with a resident pharmacist (mean 33 wards per hospital, 95% CI 23–43). There was also a non-significant difference in out-of-hours pharmacy service among NHS trusts; a non-resident on-call pharmacist was less likely to be found in foundation trusts (42% acute, 58% foundation) than a resident pharmacist (acute 22%, foundation 78%) (p = 0.42, chi-square with Yates’ correction).

**Figure 2:**
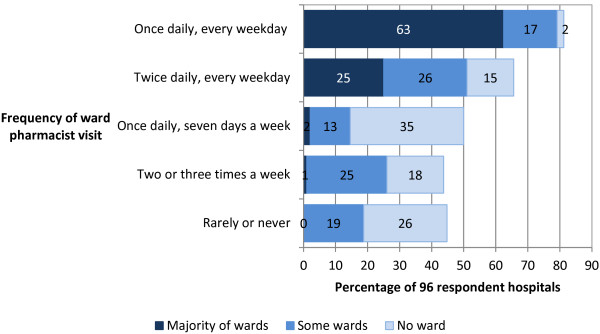
**Frequency of ward pharmacist visits in English NHS hospitals.** Totals do not sum to 100% as a number of respondents selected answers that indicated ‘majority of wards’ for a particular option and therefore the remaining options were not applicable.

**Figure 3:**
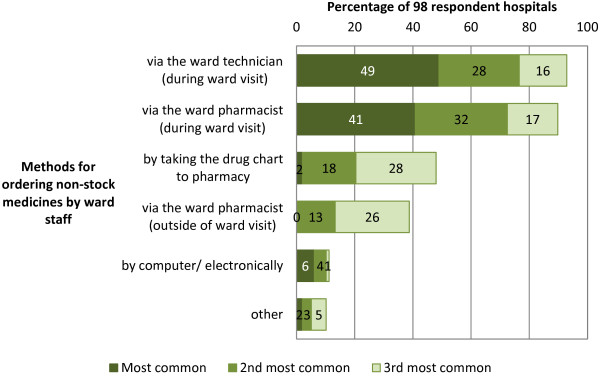
**Methods used to order non-stock medicines by ward staff in English NHS hospitals.** Totals do not sum to 100% as respondents were asked to rank the three most common methods rather than rank all methods.

A total of 96 respondents answered the question about frequency of ward pharmacist visits; the majority of hospitals (86; 90%) had at least one daily pharmacist visit on most wards every weekday (Figure 2). An overview of medication ordering methods used by respondent hospitals (both during and outside pharmacy opening hours) is presented in Table 2. As more than one method could be used in each hospital, respondents were also asked to rank the three methods that were most common during pharmacy opening hours (Figure 3). A sub-analysis of the 14 (15%) hospitals that had both a ward pharmacist and a pharmacy technician on the majority of wards revealed that the most common method for ordering medicines was via the ward pharmacist during their ward visit (6; 43%), followed by the ward technician during their ward visit (5; 36%), and one (8%) of each of: taking the drug chart to pharmacy, via the computer/electronically, and other ward-based pharmacy teams.

**Figure 4:**
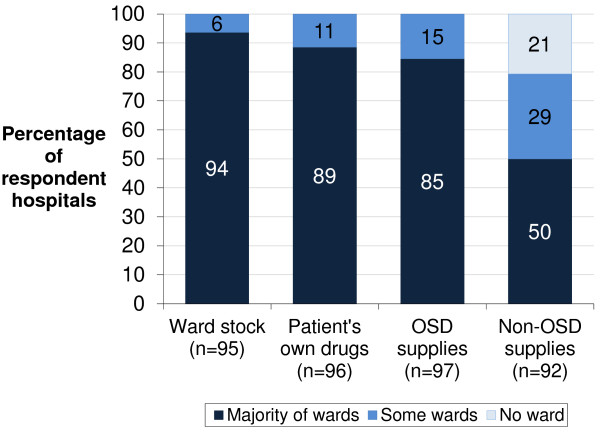
**Types of medication supply used for inpatient medication administration in English NHS hospitals.** n represents the number of complete responses for each type of medication supply. OSD, one-stop dispensing.

In general, the majority of respondent hospitals used ward-stock, OSD supplies, and PODs on the majority of medical and surgical wards (Figure 4). However, inter- and intra-hospital variation was prevalent for the use of non-OSD supplies. Three respondents additionally selected ‘other’ and specified the use of pre-labelled packs (medicines pre-labelled with standard dosage instructions but not the patient’s name) for inpatient use. None reported the use of unit dose dispensing. When asked about the most common type of medication supply used on inpatient wards, 31 (34%) respondents reported PODs, 31 (34%) reported OSD, 27 (30%) reported ward-stock, and 1 (1%) reported non-OSD supplies.

### Ward-based medication storage and transport during nurses’ drug rounds

**Figure 5:**
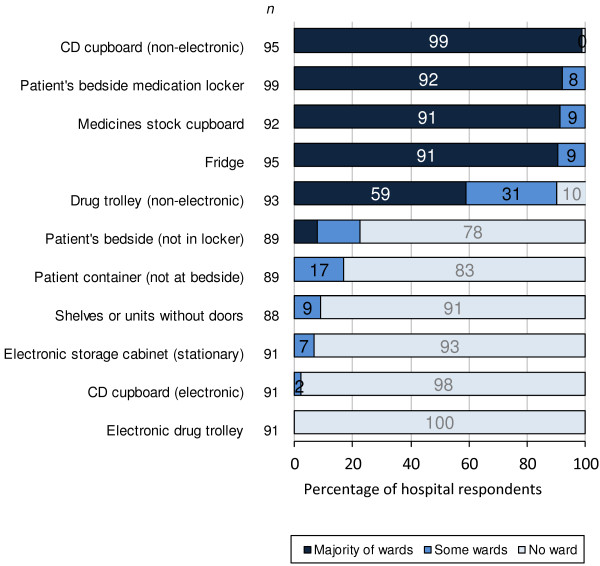
**Availability of different ward-based medication storage facilities on wards in English NHS hospitals.** n represents total number of respondent hospitals for each medication storage facility. CD: controlled drugs.

**Figure 6:**
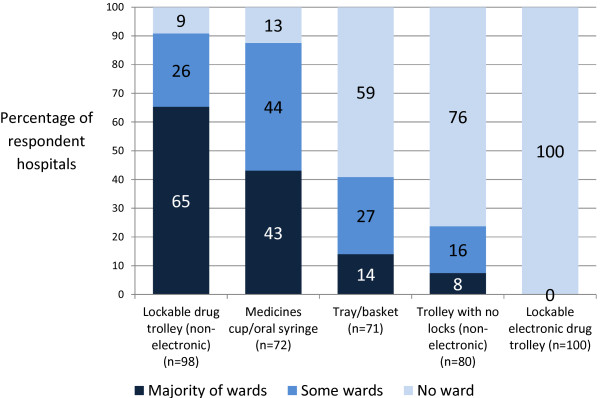
**Methods used to transport oral medicines during drug rounds in English NHS hospitals.** n represents the number of complete responses for each method used to transport oral medications to patients.

Overall, four of 11 types of medication storage facility were available on the majority of wards in respondents’ hospitals: a non-electronic controlled drugs (CD) cupboard, patient bedside medication lockers, medicines stock cupboards, and a fridge (Figure 5). Use of drug trolleys was associated with the most intra-hospital variation; 31% of respondent hospitals used these on some wards only. Exploratory analysis by SHA suggests that drug trolleys remain widely used in the South Central (all 3 respondent hospitals) and London SHAs (6 of 7 hospitals), and least used in the North West (3 of 11 hospitals) and East Midlands SHAs (2 of 7 hospitals). When asked about the most common medication storage used to retrieve medications at the time of administration, the majority (71; 72% of 98 respondents) reported patient bedside medication locker, drug trolley (15; 16%), medicines cupboards (10; 11%), and patients’ bedside table or belongings (2; 2%). There were also inter- and intra-hospital variations in the methods used to transport medicines to patients during drug rounds (Table 2 and Figure 6). Three respondents selected ‘other’ methods to transport oral medicines: “PRN (pro re nata; when required) lockers per bay” on the majority of wards in one hospital, “drugs cupboard in 6-bedded bay” on some wards in one hospital, and “individual items carried by nurse (in hands)” on some wards in one hospital.

### Medication administration processes, policies, and guidance

Respondents were asked if double-checking prior to administration was required for five specific groups of drugs: 82 (85% of 97 usable responses) stated ‘yes’ for IV medications, 63 (65%) for IV fluids, 94 (97%) for parenteral chemotherapy, 73 (75%) for oral chemotherapy and 81 (83%) for paediatric doses. Double-checking of CDs was excluded from this question as this is a legal requirement in the UK. When asked an open question about which other specific drugs required double-checking prior to administration, 37 (58% of 64 usable responses) respondents reported 15 types of drug (Table 3). The route of administration of additional named drugs was not specified by the respondents.

**Table 3:** Additional information provided by 64 respondents on specific drugs that required double-checking prior to administration

**Drug name/group**	**No of respondent hospitals (%)**
Double checking of specific drugs required but names of drugs not provided	27 (42)
Insulin	16 (25)
Heparin	7 (11)
Complex preparations	6 (9)
Potassium	5 (8)
Epidurals	3 (5)
Infusion devices	2 (3)
Oral methotrexate	2 (3)
Saline [sodium chloride 0.9%] flushes	2 (3)
Therapeutic doses of low molecular weight heparins	2 (3)
Clinical trial drugs	2 (3)
“High risk” [unspecified] intravenous drugs	1 (2)
Intravenous immunoglobulin	1 (2)
Midazolam	1 (2)
Paediatric doses requiring calculations	1 (2)

Total percentage is over 100% as some respondent hospitals had more than one drug-specific double-checking policy in place.

### Local medication safety improvement initiatives

A total of 56 (59% of 95 usable responses) respondents reported the routine use of a ‘do not disturb overall/sash’ by nursing staff during medication administration on at least one ward in their hospital. Administration of medications by a patient’s carer was routinely practised on at least one ward in 24 (27% of 89 usable responses) of respondents’ hospitals; of these, 23 were in mixed adult and paediatric hospitals and 1 was in an adult-only hospital. Overall, seven other strategies were described by 32 respondents (Table 4). The most frequently reported local initiatives were based on expanding ward pharmacy services and near-patient dispensing.

**Table 4:** Local initiatives reported in use in English NHS hospitals to improve medication safety

**Local initiative**	**Number of hospitals**	**Examples**
Extensive ward pharmacy technician and/or ward pharmacy assistant service	10	Technician discharge transcribing service
		Trial of technician medication administration
Near-patient dispensing	9	Use of mobile dispensing units, satellite dispensary, and pre-labelled packs
Extended pharmacy services to wards	7	Increased frequency of ward pharmacy visits, increased pharmacy opening hours, and provision of pharmacy service to wards on weekends
Use of OSD and PODs	6	
Self-administration schemes	4	Specific self-administration scheme for patients with Parkinson’s disease and separately for maternity units, and an ‘opt-out’ patient self-administration scheme
Technology	3	EPMA, automated medication storage cabinets (for example, Omnicell®), an electronic discharge prescribing system, and an electronic prescription tracking system
Quarterly medication storage review on wards	2	
Other	8	Director/matron walkabouts with medicines checks on wards to identify potential medication problems and provide immediate feedback to ward staff, fast-track medication request system, pneumatic tube system, non-OSD supplies being additionally labelled with “inpatient supply only” to remind staff not to issue these to patients on discharge, standard operating procedures for nurses on specific administration processes, target turnaround times for inpatient supply, and changed order of tasks during drug administrations with IVs administered first followed by medicines on a critical list then other non-IV medications.

EPMA, electronic prescribing and medication administration; IV, intravenous; OSD, one-stop dispensing; POD, patients’ own drugs.

## Discussion

### Main findings

This paper reports for the first time the prevalence of a number of core medication systems in English NHS hospitals. The majority of hospitals used paper-based prescribing (87%), patient bedside medication lockers (92%), ward stock (94%), PODs (89%), and OSD supplies (85%) in the majority of inpatient medical and surgical wards. However, hospitals varied in the methods used to order medications during pharmacy opening hours, particularly in relation to whether medicines were ordered via the ward pharmacist or a ward pharmacy technician. There were also inter- and intra-hospital variations in practices that were standard prior to the national introduction of PODs, OSD, and patient bedside medication lockers; this included use of drug trolleys to store and transport medicines, use of other methods to transport medicines during drug rounds, and the use of non-OSD supplies for inpatient use. Such variations suggest hospitals have implemented these national initiatives in different ways. Exploratory analysis by SHA suggests that there were some geographical differences in the use of drug trolleys and non-OSD supplies. In addition, we have documented the prevalence of a number of medication administration related policies, guidance and double-checking practices. Across English NHS hospitals, current efforts reported by pharmacy respondents to improve medication safety appear to concentrate on extending ward and clinical pharmacy services.

### Comparison with previous research

This is the first national survey of medication systems used in English NHS hospitals. Previous surveys, both in the UK and elsewhere, have focused only on pharmacy services [18,20,27,28] and therefore many aspects of our survey findings cannot be compared with existing literature. A recent European survey [28,29] of hospital medication procurement and distribution suggested that 37.5% of an unreported number of United Kingdom (UK) hospital pharmacies provided a unit-dose service; however it is unclear what was meant by a ‘unit-dose service’ and how this question was framed. Furthermore, the UK response rate was very low; 35% of an unreported number of questionnaires were returned and only 9% overall were usable after adjusting for unanswered questions. Comparison of our pharmacy-specific findings with those from a UK-wide clinical pharmacy survey conducted in 1992 [20], suggest that more hospital pharmacies are now providing a weekend service: 74% of UK hospitals were open on Saturdays in 1992 versus 90% of English hospitals in 2011, 10% of UK hospitals were open on Sundays in 1992 versus 74% of English hospitals in 2011. However, the percentage of hospitals that provide a resident on-call pharmacy service (9% of UK hospitals in 1992 versus 9% of English hospitals in 2011) and non-resident on-call pharmacy service (88% of UK hospitals in 1992 versus 90% of English hospitals in 2011) are similar.

### Implications for practice

Identifying similarities across the NHS provides an important context for those seeking to develop and prioritise systems-based interventions to increase medication safety. However, identifying and exploring differences between hospitals enables advantages and disadvantages of the medication systems to be better understood, and therefore inform future developments in their design, application, and/or implementation. Two of the variations we identified in medication systems were unexpected: (1) medication storage and transport (specifically relating to the use of drug trolleys), and (2) types of medication supply (specifically relating to the use of non-OSD supplies of inpatient medication).

#### Medication storage

Inter- and intra-hospital variations in drug trolley use are difficult to interpret as drug trolleys serve the two functions of storage and transport. The introduction of patient bedside medication lockers around 2001 was not explicitly intended to eliminate the use of drug trolleys; patient bedside medication lockers were advocated to facilitate inpatient self-administration and the use of PODs [21]. Furthermore, bedside medication lockers could not replace the ‘transport’ function of drug trolleys. However our survey revealed drug trolley use to be relatively low; drug trolleys have previously been reported as a standard component of medication administration during drug rounds in UK hospital inpatient wards, although with no quantitative substantiation [5,30]. Data from our survey also suggest that staff in some hospitals are using other devices to transport medications, for example, a tray or a basket, with or without a dressing trolley, to transport medications to the patient’s bedside during drug rounds. These alternative solutions may have arisen from the need to transfer medications from stock cabinets to the patient’s bedside where medication is not stored in the patient’s bedside medication locker, either due to lack of space or because it may be inefficient to store commonly used medicines in each locker The implication is that there may be a role for re-introducing lockable drug trolleys or some sort of lockable and/or wheelable device for transporting medications to the patient’s bedside on some wards. Further research is needed to identify the effects of using different devices to transport medications during drug rounds. In addition, we suggest research should also seek to identify the environmental and process-related factors associated with achieving maximum benefit from the use of different medication transport devices.

#### Types of medication supplies

Findings suggest only 50% of English hospitals now use non-OSD inpatient supplies compared with what would have been standard prior to the introduction of OSD. “[*By April 2002*] *all hospitals will have a ‘one stop dispensing/dispensing for discharge’ schemes”*. This was one of the milestones set by the Department of Health in the National Service Framework for Older People (2001), which was taken further by the Audit Commission (2001) to promote original pack dispensing and patient self-administration schemes, alongside implementation of patient bedside medication lockers. However, it was not explicit in these documents whether or not traditional ‘non-OSD’ inpatient supplies still had a role. Ten years on, our results reveal use of OSD supplies to some extent in all hospitals, and on the majority of inpatient wards in 85%. This high uptake may indicate that the potential benefits of OSD have translated into real benefits in practice; this may also explain why only 50% hospitals continue to use non-OSD inpatient labelled supplies on the majority of wards and 21% of hospitals do not use these at all. However, further research is required to substantiate these speculations and to explore the rationale for dispensing all inpatient medication as OSD supplies, even for inpatient medication which is extremely unlikely to be continued at discharge.

### Strengths and limitations

A strength of our study was the census approach. In addition we achieved a higher than previously reported response rate (61%) compared with other similar surveys in the US (40% and 29% of hospitals; Pedersen 2012; 2011, respectively), and for the UK response (35%) in a recent European survey [28]. Responses in the present study also represented a range of hospital sizes from both acute and foundation NHS trusts.

The main limitation was that we focused on English NHS hospitals and therefore the findings cannot be extrapolated elsewhere. Second, for simplicity we asked respondents to report for their main acute hospital if there was more than one in their trust; this was based on the assumption that hospitals within the same trust are likely to have the broadly the same systems and processes. Third, some parts of the questions were not completed by all respondents; however only three questions had a response rate of less than 80% and therefore unlikely to have affected interpretation of the majority of the results. These three questions asked if there were specific drugs that required a double-check prior to administration (64% of respondents answered this question), whether a tray or basket was used to transport medications on all, most, some, one or no wards (71%), and if a medicines cup or oral syringe was used on all, most, some, one, or no wards (72%). Fourth, a small number of questions asked respondents to describe use of the system ‘in their experience’; responses for these subjective questions should therefore be interpreted with care. Lastly, we did not explore the use of a number other technologies that can be used to support medication administration, for example, the use of bar-codes to verify medication administration and ‘smart’ infusion pumps. However in our experience, these are uncommon within England at present.

### Future research

In addition to the suggestions for future research around medication storage, we suggest research to explore the effects of other different medication systems and processes on MAEs and to develop potential NHS-wide interventions to reduce MAEs. Furthermore, findings from the survey may provide a useful starting point for future surveys to monitor the use of hospital medication systems and processes. The potential future expansion of EPMA will most probably lead to substantial changes. Thus, monitoring the use of different hospital medication systems will not only facilitate prioritisation of potential NHS-wide interventions to increase medication safety, but also provide an indicator of the pace of change in the NHS.

## Conclusion

In this first national survey of English hospital systems, we have described the extent of inter- and intra-hospital variation in medication systems. Such variations suggest that hospital-wide EPMA is at its infancy in the majority of hospitals, and that hospitals have implemented some core medication systems in different ways, particularly in relation to the use of ward-based medication storage and transport systems and the use of double-checking policies for specific drugs or groups of drugs. These variations may affect the generalisability of interventions to reduce MAEs, nursing staff workflow, interruptions during drug administration, and importantly, the potential for MAEs. Further research is needed to explore the implications of systems variations on MAEs directly and also indirectly.

## Competing interests

The authors declare that they have no competing interests.

## Authors’ contributions

All authors contributed to conception and study design. Questionnaire format and layout were developed by MM and ZA. Data analysis was undertaken by MM with assistance from BDF and NB. MM was responsible for interpretation of the results, with assistance from BDF, NB and ZA. The paper was drafted by MM and all authors commented critically on this before approval of the final version for submission.

## Pre-publication history

The pre-publication history for this paper can be accessed here:

http://www.biomedcentral.com/1472-6963/14/93/prepub

## Supplementary Material

Additional file 1 National survey of medication systems in English NHS hospitals. Click here for file
