# Supplementary material for: A national survey of inpatient medication systems in English NHS hospitals
Source: BMC Health Serv Res. 2014 Feb 27;14:93. doi: 10.1186/1472-6963-14-93 (PMC3943404; doi:10.1186/1472-6963-14-93)
Supplement: Additional file 1 — National survey of medication systems in English NHS hospitals. [file 1472-6963-14-93-S1.pdf]

The School of Pharmacy
University of London

Imperial College Healthcare NHS
NHS Trust

Centre for Medication Safety and Service Quality

National survey of medication systems in English NHS hospitals

Thank you for taking part in this survey.
This survey aims to identify which in-patient and discharge medication systems are currently in use across the NHS.

Your response is invaluable to us as it will contribute to the knowledge and understanding of medication systems used in the NHS, and inform future development of strategies to:
(1) reduce medication errors, (2) streamline hospital medication systems and (3) reduce wasted medications.

As a thank you for your participation, we will send you a copy of the results once the national survey is complete.

Please answer the questions in relation to the main acute hospital in your trust. If your trust has multiple acute hospitals, please choose one of these on which to base the questionnaire.
Only one questionnaire is required for each trust.

We appreciate that you might not be familiar with all the systems used in your hospital. Please complete the questionnaire as fully as you can and feel free to ask colleagues as appropriate. There is also a 'not sure' option for some questions.

Your answers will remain confidential.
The questionnaire will take approximately 20 to 30 minutes to complete.

Please return your completed questionnaire using the freepost envelope provided by **Friday 22nd July 2011**

Thank you for your time, we really appreciate it.

If you have any questions about this survey please feel free to contact us:

Monsey McLeod
monsey.mcleod@imperial.nhs.uk
TEL: 020 3313 0521
FAX: 020 3311 1342

Zamzam Ahmed
zamzam.ahmed@phd.pharmacy.ac.uk
TEL: 020 7874 1272
FAX: 020 7387 5693

PART ONE

This section is about the medication processes and resources currently in use at your hospital.

A: About your hospital

1. **What is the name of the trust that you work in?** ____

2. **How many acute hospitals are there in this trust?** __________acute hospitals

3. **What is the name of the hospital that you are answering this questionnaire for?** *Please answer the questions in relation to the main acute hospital in your trust. If your trust has multiple acute hospitals, please choose one of these on which to base the questionnaire.* ____

4. **What in-patient group(s) does this hospital treat?**
   - ☐ Adults only
   - ☐ Paediatrics only
   - ☐ Mixed adult and paediatrics

5. **Approximately how many in-patient wards are there in this hospital?** __________in-patient wards

B: Pharmacy service

For this section, please exclude any intensive care, maternity and/or mental health wards in your hospital. Please answer each statement in relation to what you see **in practice** on in-patient wards in this hospital, and not what could or should happen**. Please select one option for each part of the question, unless stated otherwise.**

6. **In general, a ward pharmacist visits the wards:**
   a. twice daily, every weekday on
      - ☐ All wards *(skip to Question 7)*
      - ☐ Most wards
      - ☐ Some wards
      - ☐ One ward
      - ☐ No wards
      - ☐ Not sure

   b. once daily, seven days a week on
      - ☐ All wards *(skip to Question 7)*
      - ☐ Most wards
      - ☐ Some wards
      - ☐ One ward
      - ☐ No wards
      - ☐ Not sure

   c. once daily, every weekday on
      - ☐ All wards *(skip to Question 7)*
      - ☐ Most wards
      - ☐ Some wards
      - ☐ One ward
      - ☐ No wards
      - ☐ Not sure

   d. two or three times a week on
      - ☐ All wards *(skip to Question 7)*
      - ☐ Most wards
      - ☐ Some wards
      - ☐ One ward
      - ☐ No wards
      - ☐ Not sure

6. **Continued.**
   e. rarely or never on
      - ☐ All wards
      - ☐ Most wards
      - ☐ Some wards
      - ☐ One ward
      - ☐ No wards
      - ☐ Not sure

   f. other (please specify):
      - ☐ All wards
      - ☐ Most wards
      - ☐ Some wards
      - ☐ One ward

7. **Typically, how many hours a day is the pharmacy open in this hospital?** (for in-patient medication supply)
   a. On weekdays _____________hours/day
   b. On Saturdays _____________hours/day
   c. On Sundays _____________hours/day

8. **When the in-patient pharmacy is closed, which of the following are available?** *(Please select all that apply)*
   a. ☐ On call pharmacist
   b. ☐ Resident pharmacist
   c. ☐ None of the above

C: Medication supply and storage on in-patient wards.

For this section, please continue to exclude any intensive care, maternity and/or mental health wards in your hospital. Please answer each statement in relation to what you see **in practice** on in-patient wards in this hospital, and not what could or should happen**. Please select one option for each part of the question, unless stated otherwise.**

9. (i) In general, in-patient wards in this hospital use:

a.	patients' own supplies (medications from home) on	☐ All wards<br>☐ Most wards<br>☐ Some wards<br>☐ One ward<br>☐ No wards<br>☐ Not sure
b.	ward stock on	☐ All wards<br>☐ Most wards<br>☐ Some wards<br>☐ One ward<br>☐ No wards<br>☐ Not sure
c.	non-stock medications labelled for in-patient use (no directions on label) on	☐ All wards<br>☐ Most wards<br>☐ Some wards<br>☐ One ward<br>☐ No wards<br>☐ Not sure
d.	one-stop dispensing supplies (directions on label for patient) on	☐ All wards<br>☐ Most wards<br>☐ Some wards<br>☐ One ward<br>☐ No wards<br>☐ Not sure
e.	other (please specify): __________________	☐ All wards<br>☐ Most wards<br>☐ Some wards<br>☐ One ward

9.(ii) In your experience, which three types of medication supplies (*a to e above*) are most commonly used on in-patient wards in your hospital? *(please enter the relevant letter from above)*

Most common: ______________

2nd most common: ______________

3rd most common: ______________

10. (i) In general, non-stock medications are ordered during pharmacy opening hours:

a.	via the ward pharmacist on their ward visit	☐ All wards<br>☐ Most wards<br>☐ Some wards<br>☐ One ward<br>☐ No wards<br>☐ Not sure
b.	via the ward pharmacy technician on their ward visit	☐ All wards<br>☐ Most wards<br>☐ Some wards<br>☐ One ward<br>☐ No wards<br>☐ Not sure
c.	by contacting the ward pharmacist outside of their ward visit	☐ All wards<br>☐ Most wards<br>☐ Some wards<br>☐ One ward<br>☐ No wards<br>☐ Not sure
d.	by taking the drug chart to pharmacy	☐ All wards<br>☐ Most wards<br>☐ Some wards<br>☐ One ward<br>☐ No wards<br>☐ Not sure
e.	by computer/ electronically	☐ All wards<br>☐ Most wards<br>☐ Some wards<br>☐ One ward<br>☐ No wards<br>☐ Not sure
f.	other method(s) (please specify): __________________	☐ All wards<br>☐ Most wards<br>☐ Some wards<br>☐ One ward

10.(ii) In your experience, which three methods (*a to f above*) are most commonly used on in-patient wards in your hospital? *(please enter the relevant letter from above)*

Most common: ______________

2nd most common: ______________

3rd most common: ______________

11. (i) In your experience, which of the following methods are used to obtain medications for in-patient wards outside of pharmacy opening hours? *(Please select all that apply)*

a. ☐ Borrow from another patient's supply on the same ward (already labelled and supplied from hospital pharmacy)
b. ☐ Borrow from another ward (ward stock)
c. ☐ Contact the on-call pharmacist
d. ☐ Obtain the medication from a reserve/emergency drug cupboard (non-electronic)
e. ☐ Obtain the medication from reserve/emergency drug cupboard (electronic)
f. ☐ Other method(s) (please specify):

____

11. (ii) In your experience, which three methods (*a to f above*) are most commonly used to obtain medications out of hours in this hospital?
(please enter the relevant letter from above)

Most common: ______________

2nd most common: ______________

3rd most common: ______________

12. In general, oral medications are prescribed on:

(i) in-patient paper drug charts	(ii) in-patient electronic prescribing system
☐ All wards	☐ All wards
☐ Most wards	☐ Most wards
☐ Some wards	☐ Some wards
☐ One ward	☐ One ward
☐ No wards	☐ No wards
☐ Not sure	☐ Not sure

13. (i) In general, oral medications on the wards are stored in:

a. medicines cupboard
☐ All wards
☐ Most wards
☐ Some wards
☐ One ward
☐ No wards
☐ Not sure

13. (i) Continued. In general, oral medications on the wards are stored in:

b. shelves or units without doors
☐ All wards
☐ Most wards
☐ Some wards
☐ One ward
☐ No wards
☐ Not sure

c. drug trolley (non-electronic)
☐ All wards
☐ Most wards
☐ Some wards
☐ One ward
☐ No wards
☐ Not sure

d. electronic drug trolley
☐ All wards
☐ Most wards
☐ Some wards
☐ One ward
☐ No wards
☐ Not sure

e. electronic storage cabinet (stationary)
☐ All wards
☐ Most wards
☐ Some wards
☐ One ward
☐ No wards
☐ Not sure

f. fridge
☐ All wards
☐ Most wards
☐ Some wards
☐ One ward
☐ No wards
☐ Not sure

g. controlled drugs cupboard (non-electronic)
☐ All wards
☐ Most wards
☐ Some wards
☐ One ward
☐ No wards
☐ Not sure

h. controlled drugs cupboard (electronic)
☐ All wards
☐ Most wards
☐ Some wards
☐ One ward
☐ No wards
☐ Not sure

i. patient's lockable bedside cabinet
☐ All wards
☐ Most wards
☐ Some wards
☐ One ward
☐ No wards
☐ Not sure

Question 13 continues on next page

13. (i) Continued. In general, oral medications on the wards are stored in:

j.	patient's bedside table or belongings	☐ All wards<br>☐ Most wards<br>☐ Some wards<br>☐ One ward<br>☐ No wards<br>☐ Not sure
k.	patient specific container located away from patient's bedside (e.g. in medication room)	☐ All wards<br>☐ Most wards<br>☐ Some wards<br>☐ One ward<br>☐ No wards<br>☐ Not sure
l.	other location(s) (please specify):<br>__________________	☐ All wards<br>☐ Most wards<br>☐ Some wards<br>☐ One ward<br>☐ No wards

13. (ii) In your experience, where would oral medications most commonly be retrieved from *(a to l above)* at the time of administration in your hospital? *(please enter the relevant letter from above)*

Most common: ______________

2nd most common: ______________

3rd most common: ______________

D: Medication administration, policies and guidance

For this section, please continue to exclude any intensive care, maternity and/or mental health wards in your hospital. Please answer each statement in relation to what you see **in practice** on in-patient wards in this hospital, and not what could or should happen. **Please select one option for each part of the question, unless stated otherwise.**

14. In general, regularly prescribed medications are administered on scheduled drug rounds on:

☐ All wards
☐ Most wards
☐ Some wards
☐ One ward
☐ No ward
☐ Not sure

15. For oral medications that are not stored at the patient's bedside, how are the medications transported to the patient from where they are stored?

a.	Lockable electronic drug trolley	☐ All wards<br>☐ Most wards<br>☐ Some wards<br>☐ One ward<br>☐ Not available<br>☐ Not sure
b.	Lockable drug trolley (non-electronic)	☐ All wards<br>☐ Most wards<br>☐ Some wards<br>☐ One ward<br>☐ Not available<br>☐ Not sure

15. Continued. For oral medications that are not stored at the patient's bedside, how are the medications transported to the patient from where they are stored?

c.	Trolley with no locks (non-electronic)	☐ All wards<br>☐ Most wards<br>☐ Some wards<br>☐ One ward<br>☐ Not available<br>☐ Not sure
d.	Tray/basket	☐ All wards<br>☐ Most wards<br>☐ Some wards<br>☐ One ward<br>☐ Not available<br>☐ Not sure
e.	Medicines cup/oral syringe	☐ All wards<br>☐ Most wards<br>☐ Some wards<br>☐ One ward<br>☐ Not available<br>☐ Not sure
f.	Other (please specify):<br>__________________	☐ All wards<br>☐ Most wards<br>☐ Some wards<br>☐ One ward<br>☐ No wards<br>☐ Not sure

16. Excluding controlled drugs, which medications, if any, require a double check at administration in your hospital? *(i.e. administration is checked by a second member of staff)*

a.	Intravenous medications	☐ Yes<br>☐ No<br>☐ Not sure
b.	Intravenous fluids	☐ Yes<br>☐ No<br>☐ Not sure
c.	Oral chemotherapy	☐ Yes<br>☐ No<br>☐ Not sure
d.	Parenteral chemotherapy	☐ Yes<br>☐ No<br>☐ Not sure
e.	Doses administered to paediatric patients	☐ Yes<br>☐ No<br>☐ Not sure
f.	Specific drugs (e.g. heparin, insulin, please list)<br>______________________<br>______________________<br>______________________<br>______________________	☐ Yes<br>☐ No<br>☐ Not sure

17. What medication administration related policies and guidance is there in your hospital?

a.	Self-administration of medications by in-patient	☐ Yes<br>☐ No<br>☐ Not sure
b.	Nil-by-mouth policy	☐ Yes<br>☐ No<br>☐ Not sure
c.	Intravenous administration guide (hard copy)	☐ Yes<br>☐ No<br>☐ Not sure
d.	Intravenous administration guide (electronic copy)	☐ Yes<br>☐ No<br>☐ Not sure
e.	Guidance on what to do if medication is not available on the ward	☐ Yes<br>☐ No<br>☐ Not sure
f.	Guidance on out of hours access to medications	☐ Yes<br>☐ No<br>☐ Not sure

18. Which of the following practices are routinely used on at least one ward in this hospital?

a.	Administration of medications by the patient's carer (e.g. parent, spouse)	☐ Yes<br>☐ No<br>☐ Not sure
b.	Nurses wear an overall/sash with "Do not disturb" or similar words during drug administration	☐ Yes<br>☐ No<br>☐ Not sure

Additional comments

19. Please tell us about any initiatives that have been implemented in your hospital to improve any of the following: pharmacy service to in-patients, medication supply and storage on in-patient wards and/or medication administration.
Please feel free to add any other comments you may have about the pharmacy service and/or medication processes in your hospital.

PART TWO

This section is about current and/or planned electronic prescribing systems at your hospital.

E: Electronic prescribing systems

Thinking about **all in-patient and discharge** services in your hospital, please answer the following questions about electronic prescribing. **In this part, please also include any systems used on intensive care, maternity and mental health wards.**

Examples of electronic prescribing systems are:

- *Comprehensive hospital wide prescribing systems (e.g. JAC, Cerner)*
- *Speciality targeted applications/software (e.g. ChemoCare, Varian)*
- *Systems relating to a specific part of the prescribing process (Electronic discharge prescribing)*

	Yes	No
**20. Does your hospital have any electronic prescribing system in use at the moment?**	☐ (continue to Question 21)	☐ (skip to Question 30)

21. Is there more than one electronic prescribing system in use in your hospital at the moment?

Yes ☐ If Yes, please insert number of systems: __________________

No ☐

Not sure ☐

22. Please insert the name of the electronic prescribing system(s) you have in the hospital.

Examples of electronic prescribing systems are:

- *Comprehensive hospital wide prescribing systems (e.g. JAC, Cerner)*
- *Speciality targeted applications/software (e.g. ChemoCare, Varian)*
- *Systems relating to a specific part of the prescribing process (Electronic discharge prescribing)*

System 1	System 2	System 3	System 4
____________	____________	____________	____________

23. How long has the system(s) been in place?	System 1	System 2	System 3	System 4
a. **<1 year**	☐	☐	☐	☐
b. **≥ 1 < 2 year**	☐	☐	☐	☐
c. **2 – 5 year**	☐	☐	☐	☐
d. **> 5 year**	☐	☐	☐	☐
e. **Not sure**	☐	☐	☐	☐

Please add any other comments you have about the questions on this page.

24. Please answer all statements from (a to c) for each system you have in place. The system you have allows:		System 1	System 2	System 3	System 4
a.	**Prescribing for in-patients**	☐ Yes<br>☐ No<br>☐ Not sure	☐ Yes<br>☐ No<br>☐ Not sure	☐ Yes<br>☐ No<br>☐ Not sure	☐ Yes<br>☐ No<br>☐ Not sure
b.	**Prescribing for discharge**	☐ Yes<br>☐ No<br>☐ Not sure	☐ Yes<br>☐ No<br>☐ Not sure	☐ Yes<br>☐ No<br>☐ Not sure	☐ Yes<br>☐ No<br>☐ Not sure
c.	**Others , please specify**	_______________	_______________	_______________	_______________

Please add any other comments you have. Insert the letter of the point you are referring to i.e. a. (comment) and name of system if necessary.

25. Please answer all statements from (a to h) for each system you have in place. The system you have is in routine use in:		System 1	System 2	System 3	System 4
a.	**Adult intensive therapy units**	☐ Not applicable<br>☐ All wards<br>☐ Some wards<br>☐ One ward<br>☐ No wards<br>☐ Not sure	☐ Not applicable<br>☐ All wards<br>☐ Some wards<br>☐ One ward<br>☐ No wards<br>☐ Not sure	☐ Not applicable<br>☐ All wards<br>☐ Some wards<br>☐ One ward<br>☐ No wards<br>☐ Not sure	☐ Not applicable<br>☐ All wards<br>☐ Some wards<br>☐ One ward<br>☐ No wards<br>☐ Not sure
b.	**Paediatric intensive therapy units**	☐ Not applicable<br>☐ All wards<br>☐ Some wards<br>☐ One ward<br>☐ No wards<br>☐ Not sure	☐ Not applicable<br>☐ All wards<br>☐ Some wards<br>☐ One ward<br>☐ No wards<br>☐ Not sure	☐ Not applicable<br>☐ All wards<br>☐ Some wards<br>☐ One ward<br>☐ No wards<br>☐ Not sure	☐ Not applicable<br>☐ All wards<br>☐ Some wards<br>☐ One ward<br>☐ No wards<br>☐ Not sure
c.	**Adult medical wards**	☐ Not applicable<br>☐ All wards<br>☐ Some wards<br>☐ One ward<br>☐ No wards<br>☐ Not sure	☐ Not applicable<br>☐ All wards<br>☐ Some wards<br>☐ One ward<br>☐ No wards<br>☐ Not sure	☐ Not applicable<br>☐ All wards<br>☐ Some wards<br>☐ One ward<br>☐ No wards<br>☐ Not sure	☐ Not applicable<br>☐ All wards<br>☐ Some wards<br>☐ One ward<br>☐ No wards<br>☐ Not sure
d.	**Adult surgical wards**	☐ Not applicable<br>☐ All wards<br>☐ Some wards<br>☐ One ward<br>☐ No wards<br>☐ Not sure	☐ Not applicable<br>☐ All wards<br>☐ Some wards<br>☐ One ward<br>☐ No wards<br>☐ Not sure	☐ Not applicable<br>☐ All wards<br>☐ Some wards<br>☐ One ward<br>☐ No wards<br>☐ Not sure	☐ Not applicable<br>☐ All wards<br>☐ Some wards<br>☐ One ward<br>☐ No wards<br>☐ Not sure
e.	**Paediatric medical wards**	☐ Not applicable<br>☐ All wards<br>☐ Some wards<br>☐ One ward<br>☐ No wards<br>☐ Not sure	☐ Not applicable<br>☐ All wards<br>☐ Some wards<br>☐ One ward<br>☐ No wards<br>☐ Not sure	☐ Not applicable<br>☐ All wards<br>☐ Some wards<br>☐ One ward<br>☐ No wards<br>☐ Not sure	☐ Not applicable<br>☐ All wards<br>☐ Some wards<br>☐ One ward<br>☐ No wards<br>☐ Not sure

Question 25 continues on next page

25. Continued. Please answer all statements from (a to h) for each system you have in place.					
**The system you have is in routine use in:**		**System 1**	**System 2**	**System 3**	**System 4**
f.	**Paediatric surgical wards**	☐ Not applicable<br>☐ All wards<br>☐ Some wards<br>☐ One ward<br>☐ No wards<br>☐ Not sure	☐ Not applicable<br>☐ All wards<br>☐ Some wards<br>☐ One ward<br>☐ No wards<br>☐ Not sure	☐ Not applicable<br>☐ All wards<br>☐ Some wards<br>☐ One ward<br>☐ No wards<br>☐ Not sure	☐ Not applicable<br>☐ All wards<br>☐ Some wards<br>☐ One ward<br>☐ No wards<br>☐ Not sure
g.	**Cancer services**	☐ Not applicable<br>☐ All wards<br>☐ Some wards<br>☐ One ward<br>☐ No wards<br>☐ Not sure	☐ Not applicable<br>☐ All wards<br>☐ Some wards<br>☐ One ward<br>☐ No wards<br>☐ Not sure	☐ Not applicable<br>☐ All wards<br>☐ Some wards<br>☐ One ward<br>☐ No wards<br>☐ Not sure	☐ Not applicable<br>☐ All wards<br>☐ Some wards<br>☐ One ward<br>☐ No wards<br>☐ Not sure
h.	**Others, please specify type of wards**	________________	________________	________________	________________

Please add any other comments you have. Insert the letter of the point you are referring to i.e. a. (comment) and name of system if necessary.

26. Which best describes your system for each statement? Please answer all statements from (a to f) for each system you have in place.					
**The system is:**		**System 1**	**System 2**	**System 3**	**System 4**
a.	**An in-house designed system** 'originally designed internally within the Trust'	☐ Yes<br>☐ No<br>☐ Not sure	☐ Yes<br>☐ No<br>☐ Not sure	☐ Yes<br>☐ No<br>☐ Not sure	☐ Yes<br>☐ No<br>☐ Not sure
b.	**Supplied by an external software supplier**	☐ Yes<br>☐ No<br>☐ Not sure	☐ Yes<br>☐ No<br>☐ Not sure	☐ Yes<br>☐ No<br>☐ Not sure	☐ Yes<br>☐ No<br>☐ Not sure
c.	**A stand alone application** 'operates without other programs'	☐ Yes<br>☐ No<br>☐ Not sure	☐ Yes<br>☐ No<br>☐ Not sure	☐ Yes<br>☐ No<br>☐ Not sure	☐ Yes<br>☐ No<br>☐ Not sure
d.	**Is linked with, or includes, the pharmacy dispensing software**	☐ Yes<br>☐ No<br>☐ Not sure	☐ Yes<br>☐ No<br>☐ Not sure	☐ Yes<br>☐ No<br>☐ Not sure	☐ Yes<br>☐ No<br>☐ Not sure
e.	**Is linked to other systems/ software in the hospital** *e.g. laboratory reports*	☐ Yes<br>☐ No<br>☐ Not sure	☐ Yes<br>☐ No<br>☐ Not sure	☐ Yes<br>☐ No<br>☐ Not sure	☐ Yes<br>☐ No<br>☐ Not sure
f.	**Is interfaced with other technologies** *e.g. bar-coding, electronic pumps.*	☐ Yes<br>☐ No<br>☐ Not sure	☐ Yes<br>☐ No<br>☐ Not sure	☐ Yes<br>☐ No<br>☐ Not sure	☐ Yes<br>☐ No<br>☐ Not sure

Please add any other comments you have. Insert the letter of the point you are referring to i.e. a. (comment) and name of system if necessary.

27. Which best describes your system for each statement? Please answer all statements from (a to o) for each system you have in place.

**The system currently offers:**		**System 1**	**System 2**	**System 3**	**System 4**
a.	**Dose checking** 'checks that dose is within normal dose range'	☐ Not applicable ☐ Yes ☐ No ☐ Not sure	☐ Not applicable ☐ Yes ☐ No ☐ Not sure	☐ Not applicable ☐ Yes ☐ No ☐ Not sure	☐ Not applicable ☐ Yes ☐ No ☐ Not sure
b.	**Dose calculations** *e.g. calculates dose per weight, calculate infusion rate, etc.*	☐ Not applicable ☐ Yes ☐ No ☐ Not sure	☐ Not applicable ☐ Yes ☐ No ☐ Not sure	☐ Not applicable ☐ Yes ☐ No ☐ Not sure	☐ Not applicable ☐ Yes ☐ No ☐ Not sure
c.	**Free text prescribing option** 'i.e. typing drug name without selecting from a list of drugs'	☐ Not applicable ☐ Yes ☐ No ☐ Not sure	☐ Not applicable ☐ Yes ☐ No ☐ Not sure	☐ Not applicable ☐ Yes ☐ No ☐ Not sure	☐ Not applicable ☐ Yes ☐ No ☐ Not sure
d.	**Drug interaction alerts**	☐ Not applicable ☐ Yes ☐ No ☐ Not sure	☐ Not applicable ☐ Yes ☐ No ☐ Not sure	☐ Not applicable ☐ Yes ☐ No ☐ Not sure	☐ Not applicable ☐ Yes ☐ No ☐ Not sure
e.	**Multi level control for prescribers** 'different levels of authority tailored per prescriber'	☐ Not applicable ☐ Yes ☐ No ☐ Not sure	☐ Not applicable ☐ Yes ☐ No ☐ Not sure	☐ Not applicable ☐ Yes ☐ No ☐ Not sure	☐ Not applicable ☐ Yes ☐ No ☐ Not sure
f.	**Prescribing by selecting a drug from a drop down (or similar) menu**	☐ Not applicable ☐ Yes ☐ No ☐ Not sure	☐ Not applicable ☐ Yes ☐ No ☐ Not sure	☐ Not applicable ☐ Yes ☐ No ☐ Not sure	☐ Not applicable ☐ Yes ☐ No ☐ Not sure
g.	**Access to drug management information** *e.g. BNF, policies, guidelines, formulary*	☐ Not applicable ☐ Yes ☐ No ☐ Not sure	☐ Not applicable ☐ Yes ☐ No ☐ Not sure	☐ Not applicable ☐ Yes ☐ No ☐ Not sure	☐ Not applicable ☐ Yes ☐ No ☐ Not sure
h.	**Allergy checker** *e.g. electronic alert appears on screen*	☐ Not applicable ☐ Yes ☐ No ☐ Not sure	☐ Not applicable ☐ Yes ☐ No ☐ Not sure	☐ Not applicable ☐ Yes ☐ No ☐ Not sure	☐ Not applicable ☐ Yes ☐ No ☐ Not sure
i.	**Orders laboratory investigations**	☐ Not applicable ☐ Yes ☐ No ☐ Not sure	☐ Not applicable ☐ Yes ☐ No ☐ Not sure	☐ Not applicable ☐ Yes ☐ No ☐ Not sure	☐ Not applicable ☐ Yes ☐ No ☐ Not sure
j.	**Displays laboratory results**	☐ Not applicable ☐ Yes ☐ No ☐ Not sure	☐ Not applicable ☐ Yes ☐ No ☐ Not sure	☐ Not applicable ☐ Yes ☐ No ☐ Not sure	☐ Not applicable ☐ Yes ☐ No ☐ Not sure
k.	**Drug stock checking** 'checks if formulary drugs are available or out of stock'	☐ Not applicable ☐ Yes ☐ No ☐ Not sure	☐ Not applicable ☐ Yes ☐ No ☐ Not sure	☐ Not applicable ☐ Yes ☐ No ☐ Not sure	☐ Not applicable ☐ Yes ☐ No ☐ Not sure
l.	**Discharge/transfer summaries**	☐ Not applicable ☐ Yes ☐ No ☐ Not sure	☐ Not applicable ☐ Yes ☐ No ☐ Not sure	☐ Not applicable ☐ Yes ☐ No ☐ Not sure	☐ Not applicable ☐ Yes ☐ No ☐ Not sure

Question 27 continues on next page

27. Continued. Which best describes your system for each statement? Please answer all statements from (a to o) for each system you have in place.					
**The system currently offers:**		**System 1**	**System 2**	**System 3**	**System 4**
m.	**Prompts drug administration by nursing staff**	☐ Not applicable<br>☐ Yes<br>☐ No<br>☐ Not sure	☐ Not applicable<br>☐ Yes<br>☐ No<br>☐ Not sure	☐ Not applicable<br>☐ Yes<br>☐ No<br>☐ Not sure	☐ Not applicable<br>☐ Yes<br>☐ No<br>☐ Not sure
n.	**Records drug administration**	☐ Not applicable<br>☐ Yes<br>☐ No<br>☐ Not sure	☐ Not applicable<br>☐ Yes<br>☐ No<br>☐ Not sure	☐ Not applicable<br>☐ Yes<br>☐ No<br>☐ Not sure	☐ Not applicable<br>☐ Yes<br>☐ No<br>☐ Not sure
o.	**If there is any other key features of the system, please specify:**	_______________<br>_______________	_______________<br>_______________	_______________<br>_______________	_______________<br>_______________

Please add any other comments you have. Insert the letter of the point you are referring to i.e. a. (comment) and name of system if necessary.

28. Which best describes your system for each statement? Please answer all statements from (a to d) for each system you have in place.					
**On the current system, can the following be prescribed?**		**System 1**	**System 2**	**System 3**	**System 4**
a.	**Continuous Intravenous infusions (IVIs)**	☐ Not applicable<br>☐ Yes<br>☐ No<br>☐ Not sure	☐ Not applicable<br>☐ Yes<br>☐ No<br>☐ Not sure	☐ Not applicable<br>☐ Yes<br>☐ No<br>☐ Not sure	☐ Not applicable<br>☐ Yes<br>☐ No<br>☐ Not sure
b.	**Sliding scale insulin**	☐ Not applicable<br>☐ Yes<br>☐ No<br>☐ Not sure	☐ Not applicable<br>☐ Yes<br>☐ No<br>☐ Not sure	☐ Not applicable<br>☐ Yes<br>☐ No<br>☐ Not sure	☐ Not applicable<br>☐ Yes<br>☐ No<br>☐ Not sure
c.	**Warfarin**	☐ Not applicable<br>☐ Yes<br>☐ No<br>☐ Not sure	☐ Not applicable<br>☐ Yes<br>☐ No<br>☐ Not sure	☐ Not applicable<br>☐ Yes<br>☐ No<br>☐ Not sure	☐ Not applicable<br>☐ Yes<br>☐ No<br>☐ Not sure
d.	**Tapering doses** *e.g. corticosteroids*	☐ Not applicable<br>☐ Yes<br>☐ No<br>☐ Not sure	☐ Not applicable<br>☐ Yes<br>☐ No<br>☐ Not sure	☐ Not applicable<br>☐ Yes<br>☐ No<br>☐ Not sure	☐ Not applicable<br>☐ Yes<br>☐ No<br>☐ Not sure

Please add any other comments you have. Insert the letter of the point you are referring to i.e. a. (comment) and name of system if necessary.

29. Which drugs (if any) are prescribed on a supplementary paper drug chart? (Please select all that apply)

**System 1**	**System 2**	**System 3**	**System 4**
☐ None	☐ None	☐ None	☐ None
☐ Continuous IVIs	☐ Continuous IVIs	☐ Continuous IVIs	☐ Continuous IVIs
☐ Insulin	☐ Insulin	☐ Insulin	☐ Insulin
☐ Warfarin	☐ Warfarin	☐ Warfarin	☐ Warfarin
☐ Tapering doses	☐ Tapering doses	☐ Tapering doses	☐ Tapering doses
☐ Other (please specify)	☐ Other (please specify)	☐ Other (please specify)	☐ Other (please specify)
________________	________________	________________	________________

Please add any other comments you have. Insert the name of the system if necessary.

30. Does your hospital intend to introduce a new prescribing system(s)? If Yes, when:

No	<1 year	1-2 years	>2 year	Not sure
☐	☐	☐	☐	☐

Please add any other comments you have.

31. Please provide your details below if you are happy for us to contact you in case any of your responses require further clarification. Your contact information will remain confidential.

Name: ____

Role/job title: ____________________________________

Email address: ____________________________________

Phone number: ____________________________________

Bleep number: ____________________________________

32. Thank you for completing this survey. The information you have provided will help us to identify what medication systems are currently in use and will contribute to the development of future strategies to (1) reduce medication errors, (2) streamline hospital medication systems and (3) reduce wasted medications. Would you be willing to be contacted for the next stage of our research?

☐ Yes ☐ No
